# Vascular Regulation by Super Enhancer-Derived LINC00607

**DOI:** 10.3389/fcvm.2022.881916

**Published:** 2022-06-28

**Authors:** Kiran Sriram, Yingjun Luo, Dongqiang Yuan, Naseeb Kaur Malhi, Alonso Tapia, Vishnu Amaram Samara, Rama Natarajan, Zhen Bouman Chen

**Affiliations:** ^1^Department of Diabetes Complications and Metabolism, Arthur Riggs Diabetes and Metabolism Research Institute, Duarte, CA, United States; ^2^Irell and Manella Graduate School of Biological Sciences, Beckman Research Institute of City of Hope, Duarte, CA, United States

**Keywords:** enhancer, long non-coding RNA, endothelial cells, vascular smooth muscle cells, RNA-Seq, single-cell RNA-seq, diabetes, endothelial dysfunction

## Abstract

Vascular endothelial cells (ECs) play a pivotal role in whole body homeostasis. Recent advances have revealed enhancer-associated long non-coding RNAs (lncRNAs) as essential regulators in EC function. We investigated LINC00607, a super enhancer-derived lncRNA (SE-lncRNA) in human arteries with an emphasis on ECs. Based on public databases and our single cell RNA-sequencing (scRNA-seq) data from human arteries collected from healthy and diabetic donors, we found that LINC00607 is abundantly expressed in the arteries and its level is increased in diabetic humans. Using RNA-sequencing, we characterized the transcriptomes regulated by LINC00607 in ECs and vascular smooth muscle cells (VSMCs) and in basal and diabetic conditions in ECs. Furthermore, through transcriptomic and promoter analysis, we identified c-Myc as an upstream transcription factor of LINC00607. Finally, using scRNA-seq, we demonstrated that modified antisense oligonucleotide inhibitor of LINC00607 can reverse dysfunctional changes induced by high glucose and TNFα in ECs. Collectively, our study demonstrates a multi-pronged approach to characterize LINC00607 in vascular cells and its gene regulatory networks in ECs and VSMCs. Our findings provide new insights into the regulation and function of SE-derived lncRNAs in both vascular homeostasis and dysfunction in a cell-type and context-dependent manner, which could have a significant impact on our understanding of epigenetic regulation implicated in cardiovascular health and diseases like diabetes.

## Introduction

Vascular endothelial cells (ECs) at the critical interface of circulating blood and vessel wall are fundamental gatekeepers for cardiovascular health and whole-body homeostasis. These cells are sensitive to alterations in flow dynamics, nutrients and metabolites, inflammatory signals, etc. Their maladaptation to these stimuli causes EC dysfunction or endotheliopathy, which is one of the early cellular processes contributing to a variety of metabolic and cardiovascular diseases (CVDs). For example, upon exposure to chronic hyperglycemia and inflammation prevalent in diabetes, one of the biggest risk factors driving CVDs, ECs can activate a transcriptional program that decreases nitric oxide (NO) bioavailability, impairs angiogenesis, and activates pro-inflammatory and pro-fibrotic states (1, 2). Lines of evidence suggest that hyperglycemic insults can result in long-lasting disease-driving epigenetic changes including DNA methylation, histone modifications, and long non-coding RNAs (lncRNAs)-mediated modulations (3–8). Thus, better understanding of the epigenetic (dys) regulation in ECs can provide valuable information to develop improved treatment of these diseases.

LncRNAs are >200 bp in length, polyadenylated, and often spliced. A significant portion of lncRNAs is transcribed from enhancers, the major gene-regulatory elements that promote gene transcription through *cis*-action. These lncRNAs have been termed enhancer-associated lncRNAs or elncRNAs (9–12). As such, the genomic regions of elncRNAs are typically marked by permissive histone marks such as H3K27ac (active enhancers) and H3K4me1 (poised enhancers) and bound by transcription factors (TFs) (13–16). Akin to the role of enhancers to augment gene expression, elncRNAs have been suggested to indicate enhancer activity and play a locus-dependent or independent role in the regulation of gene expression *in cis* or *trans* (11, 12). In ECs, several elncRNAs have been identified to regulate various aspects of EC function. We have identified *LEENE* (*LINC00520*) that promotes the nascent RNA synthesis of eNOS, an EC hallmark gene and thereby EC homeostasis (17). GATA6-AS1 is another elncRNA shown to be hypoxia-induced and regulate angiogenesis in ECs by interacting with LOXL2 protein (18). More recently, *AERRIE* (*LINC01013*) has been reported as a flow-inducible elncRNA that modulates EC barrier function and migration (19). Most of these elncRNAs are enriched in the nucleus, bound to chromatins, and regulate transcriptional activity.

When present in stretches and clusters, enhancers have been identified as super-enhancers (SEs), which are collectively bound by an array of transcription factors (TFs) to drive transcription of genes involved in cell identity and fate (20, 21). Furthermore, SEs are enriched in single nucleotide polymorphisms (SNPs) associated with specific diseases, in a cell type-specific manner (20, 22). In ECs, enhancers and SEs not only govern EC lineage and identity, but the aberrant activation of certain SEs, e.g., through TNFα-NF-κB signaling, can also contribute to inflammation and atherogenesis (23, 24), highlighting a pivotal role of SEs in cardiovascular disease. Like enhancers, SEs can also give rise to lncRNAs. However, other than lncRNA MALAT1, which is transcribed from a universal SE and has been studied in most cell types including ECs (25–27), there are no studies investigating SE-derived lncRNAs in ECs and very few in the cardiovascular system. In our previous study to profile RNA-genome interactions in ECs treated with a combination of high glucose and TNFα (HT) mimicking the diabetic milieu, we found that the RNA-DNA interactions were highly enriched in SEs (28). Specifically, we identified the SE-derived LINC00607 whose expression was induced by HT in ECs. In HT-treated ECs, inhibition of nuclear localized LINC00607 leads to decreased monocyte adhesion and suppressed expression of pro-inflammatory gene such as PAI1 (encoded by *SERPINE1* gene), a key driver of insulin resistance and EC dysfunction (28). These findings suggest that LINC00607 may be a novel epigenetic regulator in the vasculature that plays crucial role in vascular health and disease.

A recent study identified LINC00607 as one of the most significantly upregulated genes in osteosarcoma as compared to adjacent non-cancerous tissues and demonstrated a role for this lncRNA in promoting cancer cell proliferation and invasion (29). However, the distribution, regulation, and function of LINC00607 in non-cancerous tissues, particularly in the vasculature remains to be explored. Thus, in this study we determined the expression pattern of LINC00607 in human arteries, its upstream TF, and its regulatory role in ECs under conditions pertinent to vascular homeostasis and dysfunction. Leveraging multi-omics data, we identified LINC00607 as a vascular-enriched SE-derived lncRNA that may play both *cis*- and *trans*-regulatory roles in modulating EC as well as vascular smooth muscle cell (VSMC) functions. At transcriptional level, LINC00607 is in part regulated by c-Myc, a well-known TF that regulates cellular function essential to both health and disease. Importantly, inhibition of LINC00607 leads to a profound reversal of HT-induced ECM remodeling and pro-inflammatory and pro-fibrotic activation in ECs. Together, our findings highlight LINC00607 as an emerging crucial epigenetic regulator in the vessel wall and the potential of targeting SE-derived lncRNAs such as LINC00607 for improved treatment or reversal of CVDs and metabolic memory in diabetes-associated vascular complications.

## Materials and Methods

### Human Tissues

Human tissue studies were conducted on deidentified specimens obtained from the Southern California Islet Cell Resource Center at City of Hope. The research consents for the use of postmortem human tissues were obtained from the donors' next of kin and ethical approval for this study was granted by the Institutional Review Board of City of Hope (IRB #01046). T2DM was identified based on diagnosis in the donors' medical records as well as the percentage of glycated hemoglobin A1c (HbA1c) of 6.5% or higher. The intimal RNA was isolated from human mesenteric artery by flushing the inner lumen with TRIzol once as previously described (30). Donor information is summarized in Supplementary Table 1.

### Cell Lines

For most *in vitro* experiments involving ECs, human umbilical vein ECs, i.e., HUVECs (Cell Applications Inc., San Diego, CA, Catalog # 200p-05n and lot # 3,363 and 2,914) between passages 5–8 pooled from multiple donors were used after testing negative for mycoplasma contamination. The cells were cultured in M199 (Sigma M2520) supplemented with 15% FBS (Hyclone, SH30910.02), β-endothelial cell growth factor (Sigma, E1388), and 100 units/ml penicillin and 100 mg/ml streptomycin (Thermo Fisher Scientific). Human VSMC (HVSMC) were purchased from ATCC (Manassas, VA, USA) (PCS-100-012TM) and cultured in M231 medium with smooth muscle growth supplement (Gibco, Waltham, MA, USA) as previously described (31). All the cells were maintained at 37°C with 5% CO_2_ in a hydrated incubator.

### Cell Transfection and Stimuli

For LINC00607-knockdown experiments, an antisense locked nucleic acid (LNA) modified oligonucleotide (GapmeR) specifically targeting LINC00607 (NR_037195.1) was designed by QIAGEN (28). For LINC00607-KD under basal condition, the final concentration of LINC00607-LNA is 50 nM, treated for 48 h. For LINC00607-KD in HUVEC under HT, the final concentration of LINC00607-LNA is 25 nM, treated for 72 h. For Myc-knockdown experiments, a pool of 3 different siRNAs were purchased from Thermo Fisher Scientific. c-Myc-siRNA was used at 20 nM treated for 72 h. LNA GapmeR or siRNAs were transfected into HUVECs with Lipofectamine RNAiMAX following the protocol provided by the manufacturer (Thermo Fisher Scientific). The cells were returned to M199 complete medium 4–6 h after transfection and incubated for 3 days or treated with high glucose (25 mM final concentration) + TNFα treatment (5 ng/ml; Thermo Fisher Scientific Cat # PHC3011) (abbreviated as HT) for 3 days as in our previous study (28). For the reversal experiment, ECs were treated with HT first before transfection. D-mannitol at 25 mM was used as an osmolarity control. HVSMC transfection was done as previously described (31).

### Immunoblotting

Cell lysates were resolved on an SDS-PAGE gel followed by immunoblotting with two different antibodies against c-Myc. Recombinant Anti-c-Myc antibody (ab32072, Abcam, 1:1000 dilution) or c-Myc Rabbit mAb (18,583, Cell Signaling Technology, 1:1000 dilution) were used as primary antibodies, and anti-rabbit (7,074 s, Cell Signaling Technology, 1:5000 dilution) was used as the secondary antibody. β-actin Rabbit mAb (8,457 s, Cell Signaling Technology, 1:1000 dilution) was used for loading control.

### Tube Formation Assay

Tube formation assay was performed as previously described (32) with slight modifications. Briefly HUVECs were plated on a Matrigel (Corning, Catalog # 356234) coated 24-well plate in M199 complete media, incubated for up to 12 h in 5% CO_2_ at 37°C, and examined for capillary tube formation under an inverted microscope and imaged. Three randomly selected views were captured, and the formed tubes were counted.

### Quantitative PCR, Bulk RNA-Seq, and Data Analysis

Total RNA was extracted using TRIzol reagent and cDNAs were synthesized using the PrimeScript™ RT Master Mix containing both Oligo-dT and random hexamer primers. qPCR was performed with Bio-Rad SYBR Green Supermix following the manufacturer's protocol on the Bio-Rad CFX Connect Real Time system with β-actin as internal control. All primer sequences used for qPCR amplification are listed in Supplementary Table 2.

For HUVEC bulk RNA-seq, 500 ng total RNA per sample was subjected to library construction using KAPA mRNA HyperPrep Kit (Roche Diagnostics) following the manufacturer's manual. The libraries were sequenced in HiSeq2500 using the SR50 mode. For HVSMC bulk RNA-seq, 200 ng total RNA per sample was subjected to library construction using KAPA mRNA HyperPrep Kit (Roche Diagnostics) following the manufacturer's manual. The libraries were sequenced in HiSeqX using the PE150 mode.

To analyze abundance of LINC00607, ENCODE RNA-seq data (corresponding to Supplementary Figure 2) were downloaded from GSE78534, GSE78528, GSE78548, GSE78541, GSE88601, GSE78645, GSE78660, GSE88615, GSE78671, GSE78609, GSE78613, GSE78588, GSE78540, GSE78533, GSE78536, GSE88167, GSE90268, GSE90272, GSE78619, GSE78618, GSE78649, and GSE78587.

RNA-seq data were analyzed as previously described (28, 33). The FATSQ files were aligned to human reference genome (Ensembl GRCh38.105) using STAR (v2.4.7a) (34). FeatureCounts from Subread package (v2.3.0) (35) were used to generate counts of the uniquely mapped reads. The raw count matrices were prefiltered to remove genes with very low read counts and used as input for differential gene expression analysis using the R package DESeq2 (36). Differentially expressed genes (DEGs) with *P*-values < 0.01 were considered significant and genes with Log_2_ fold change (FC) > |1| were used for subsequent analysis. Gene ontology (GO) enrichment analysis was performed using the Gene Ontology Consortium platform (37) and Benjamini-Hochberg corrected *P*-values < 0.05 were considered significantly enriched pathways.

### Single-Cell RNA-Seq Sample Preparation and Analysis

Our published datasets from EC-enriched human mesenteric arterial cells (28) was first analyzed using Cellranger (v6.1.1), the standard pipeline provided by 10X Genomics, which aligned the raw reads to human hg38 reference transcriptome. The aligned data was then processed using the R package Seurat (v3.2.3) following published guidelines (38). Initially, well-established filtering steps were performed to remove genes expressed in <3 cells and cells expressing <200 genes. A threshold was set at the 98th percentile for gene distribution in each sample, and cells above that threshold were removed to account for possible multiplets. Furthermore, cells containing >20% mitochondrial genes were filtered out. Next, the “sctransform” normalization strategy was used where the residuals of negative binomial regression were used to model each gene. Any gene expressing a positive residual indicated that more unique molecular identifiers (UMIs) were observed than predicted considering the sequencing depth and gene average expression. The converse is represented by a negative residual (39). Datasets from the four samples then underwent integration to enable joint dimensionality reduction and clustering without being influenced by donor condition. Cells-pairs in matched biological states between samples were identified as “anchors” and used to integrate the data. The standard pre-processing step of scaling was performed prior to principal component analysis (PCA) on all cells and top 1,000 highly variable genes using the scaled z-score expression values were identified. The top 15 significant principal components (PCs) were used as an input to the uniform manifold approximation and projection (UMAP) algorithm with 0.5 resolution (Seurat default). Cell clusters were generated and annotated using the following markers based on existing literature and PanglaoDB (40–42): for VSMCs, *ACTA2, MYH11, WT1*, and *TAGLN*; for ECs, *VWF, PECAM1, EGFL7, ID3, GNG11, MCAM, CLDN5*, and *CDH5*; for macrophages, *CD68, MARCH1*, and *CD52*; for fibroblasts, *LUM* and *DCN*; for T cells, *CD3D* and *CD3E*; and for NK cells, *KLRD1* and *NKG7*.

Differential expression analysis was performed on UMI counts that had been normalized using “LogNormalize” and scaled. The Seurat default, non-parametric Wilcoxon test was used to analyze genes expressed in at least 10% of cells. The log fold-change of the average expression was thresholded at 0.25 and a pseudocount of 1 was added to the averaged expression values.

For scRNA-seq of HUVECs, ECs were trypsinized and suspended into a single cell suspension for scRNA-seq library preparation following the 10x Genomics Chromium 3' expression protocol and as described (28). The same steps were utilized to perform scRNA-seq analysis on HUVECs with the following exceptions: (1) Seurat (v.4.0) R package was utilized, (2) the top 30 significant PCAs were used as input for UMAP, (3) high mitochondrial genes were defined as >25% per cell and filtered out, (4) clusters were annotated as either LINC00607 high (>1), mid (0.5–1) or low (<0.5).

### Single-Molecule RNA Fluorescent *in situ* Hybridization (smFISH) and Image Analysis

Fresh frozen human mesenteric artery cryosections of 10 μm thickness were subjected to smFISH using RNAscope™ Multiplex Fluorescent V2 Assay (ACDBio, 323100) with probes specifically designed to target the LINC00607 transcript NR_037195.1 (RNAscope® Probe—Hs-LINC00607-O1; Catalog # 894351). Briefly, the cryosections were ethanol dehydrated and permeabilized with Protease Plus reagent for 30 min before probe hybridization, signal amplification, and development following the manufacturer's protocol. smFISH in HUVECs was performed similarly with a slight modification. Briefly, cells were grown and treated on glass coverslips coated with Poly-lysine and fibronectin and then fixed with 4% paraformaldehyde. After fixing, the cells were subjected to ethanol dehydration, hydrogen peroxide pre-treatment, permeabilization with Protease III reagent (1:10 dilution) and probe hybridization before developing the signal. Image analysis was performed using FIJI (ImageJ software). For smFISH in HUVECs, LINC00607 signal per nucleus was computed by drawing a mask around the nucleus (with DAPI channel as reference) and determining the average gray value of LINC00607 signal within this mask per nucleus.

### Statistical Analysis

For all the high-throughput sequencing experiments, 2–3 biological replicates were used. For other experiments at least three independent experiments were performed. For these experiments, statistical analysis was performed using *t*-test (unpaired, two-sided) between two groups or ANOVA followed by Bonferroni *post-test* for multiple-group comparisons. *P* < 0.05 was considered as statistically significant unless otherwise specified. The quantification and statistical analysis have also been specified and detailed in the results, figure legends, and methods.

### Data Availability

All high-throughput sequencing data supporting the current study have been deposited on GEO (Accession# GSE197956). The sample information is provided in Supplementary Table 4. The bulk RNA-seq data for LINC00607-KD in HUVEC under HT is included in Accession# GSE135357.

## Results

### Expression of LINC00607 in Human Arteries

**Figure 1:**
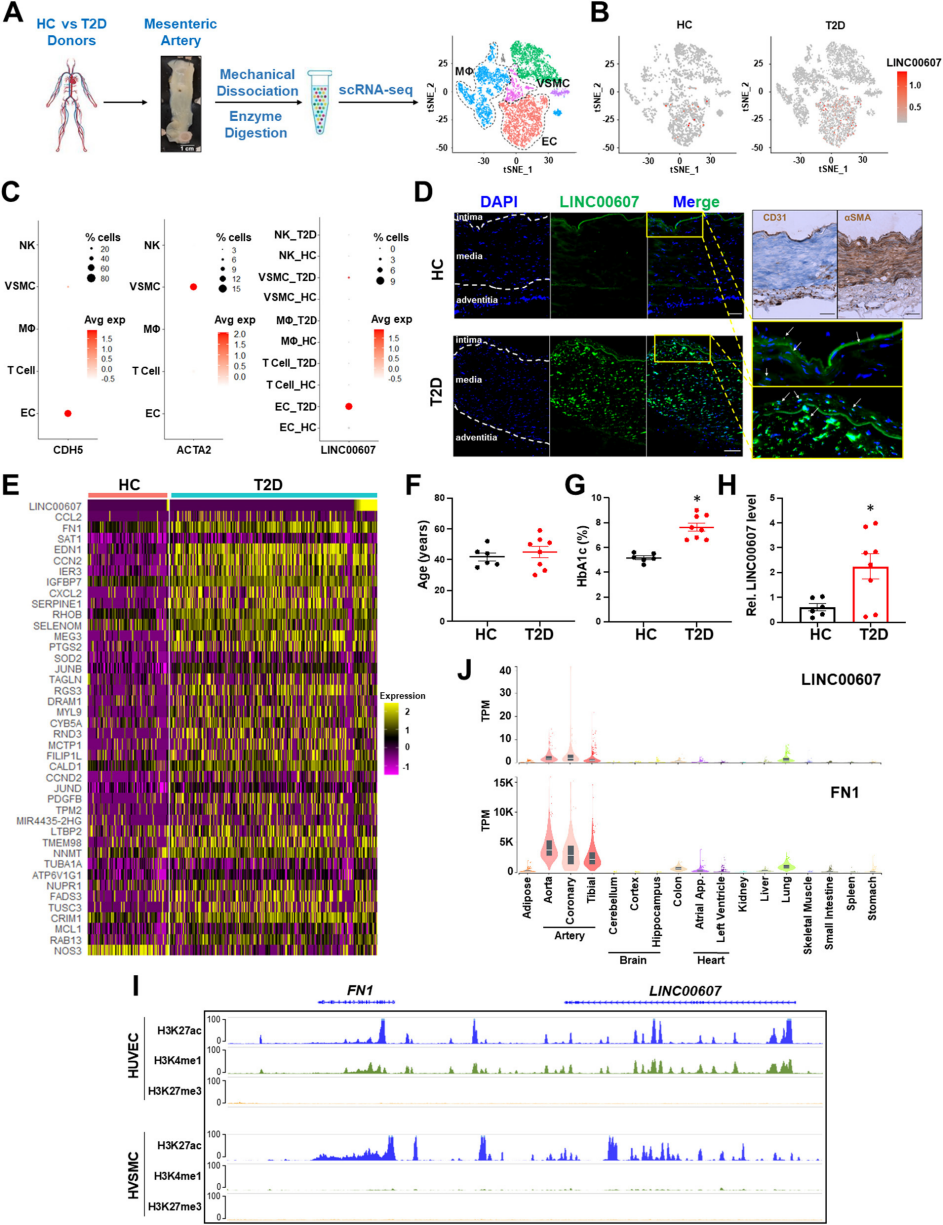
Expression of LINC00607 in human arteries. **(A)** Workflow of intimal layer isolation from human mesenteric artery samples from healthy control (HC) and type 2 diabetic (T2D) donors through single cell isolation and transcriptomic profiling to identify major cell types plotted on UMAP. **(B)** UMAP of LINC00607 expression level in each single cell across different cell clusters/cell types in HC or T2D donors. The RNA levels are represented by log-normalized unique molecular identifier (UMI) counts. **(C)** Dotplot of CDH5, ACTA2 and LINC00607 expression. Dot size corresponds to proportion of cells within the group expressing each gene, and dot color corresponds to its expression level. **(D)** smFISH of HC and T2D human mesenteric arteries with LINC00607 probe (green). Yellow boxes are zoomed in images of intima. DAPI staining indicative of nuclei. IHC with EC marker, CD31 and VSMC marker, αSMA to determine histology of vessel and delineate vessel layers. Scale bar = 100μm. **(E)** Expression heatmap of representative DE genes induced in T2D vs. HC donor-derived ECs including *LINC00607, CCL2*, and *SERPINE1*. **(F–H)** Age **(F)** and HbA1c levels **(G)** of human donors whose mesenteric arterial intima were used for LINC00607 quantification using qPCR **(H)**. Data are represented as mean ± SEM in the scatter plots with indicated *P*-value based on unpaired *t*-tests between the healthy and T2D donors. *P < 0.05 based on unpaired *t*-tests between HC and T2D donors. **(I)** ChIP-seq tracks of H3K27ac, H3K4me1, and H3K27me3 of LINC00607 and FN1 genomic loci in HUVEC and HVSMC plotted using Epigenome Browser. Data source: HUVEC H3K27ac (ENCFF000BRY), H3K4me1 (ENCFF000BST), and H3K27me3 (ENCFF135HPM); VSMC H3K27ac (ENCFF291XEQ), H3K4me1 (ENCFF013AWX), and H3K27me3 (ENCFF360FGJ). **(J)** LINC00607 and FN1 expression in human tissues revealed by the GTEx database. Figure 1A is adapted from (43).

To map the expression of LINC00607 in human arteries, we leveraged the scRNA-seq data previously obtained from mesenteric arteries of 4 human donors (2 healthy control vs. 2 T2D) (28). Specifically, the arterial tissues were collected using a published method in which the intimal layer was dissociated, followed by enzymatic digestion into single-cell suspension for 10x Genomics scRNA-seq workflow (43). Analysis of >12,000 single cells identified 3,366 ECs and 1,120 VSMC among other cells present in the mesenteric artery (Figure 1A). LINC00607 was detected in ECs and VSMCs by scRNA-seq, which captures transcripts with poly A tails (Figures 1B,C). scRNA-seq analysis also revealed higher levels of LINC00607 in T2D ECs and VSMCs as compared to healthy donors, evident by both average expression levels in single cells and percentage of cells expressing LINC00607 (Figures 1B,C). As cell type controls, *CDH5* (encoding vascular endothelial cadherin) and *ACTA2* (encoding alpha smooth muscle cell actin) were abundantly expressed in ECs and VSMCs, respectively.

Consistent with scRNA-seq, smFISH revealed the expression of LINC00607 in intima and media of the mesenteric arteries, with remarkably higher signals in the T2D donor as compared to the healthy control (Figure 1D), whereas negative controls yielded no significant signals in either tissue (Supplementary Figure 1). Of note, as a validation of the diabetic state of our select donor-derived tissues, expression levels of many well-known EC dysfunction markers (e.g., *CCL2, FN1, SERPINE1, EDN1*, etc.) were increased in ECs of T2D vs. control donors (Figure 1E). We also verified the expression pattern of LINC00607 by qPCR using tissues collected from multiple donors. Indeed, LINC00607 levels in the intima-enriched arterial tissues were significantly higher in age-matched T2D donors (*n* = 8) relative to those in the healthy individuals (*n* = 6) (Figures 1F–H).

We exploited multiple transcriptomic and epigenomic datasets available at ENCODE and genotype–tissue expression (GTEx) (44, 45) to determine the expression pattern of LINC00607. Based on ChIP-seq data, the genomic region of *LINC00607* is indeed highly enriched in H3K27ac and H3K4me1 in HUVECs and VSMCs, indicating an enhancer state in these two cell types (Figure 1I). Furthermore, LINC00607 RNA is transcribed in arterial ECs and VSMCs evident by RNA-seq data from multiple ENCODE datasets (Supplementary Figure 2). Interestingly, FN1, one of the best characterized genes associated with CVD, is located ~175Kb downstream of LINC00607 (Figure 1I). According to the tissue distribution revealed in GTEx, the highest abundance of LINC00607, like that of FN1, was detected in arteries, as compared to other major organs and tissues (Figure 1J). Taken together, these data suggest that LINC00607 is a vascular-enriched SE-derived lncRNA expressed in ECs and VSMCs and is upregulated in diabetes.

### LINC00607 Regulates Basal Function in ECs and VSMCs

**Figure 2:**
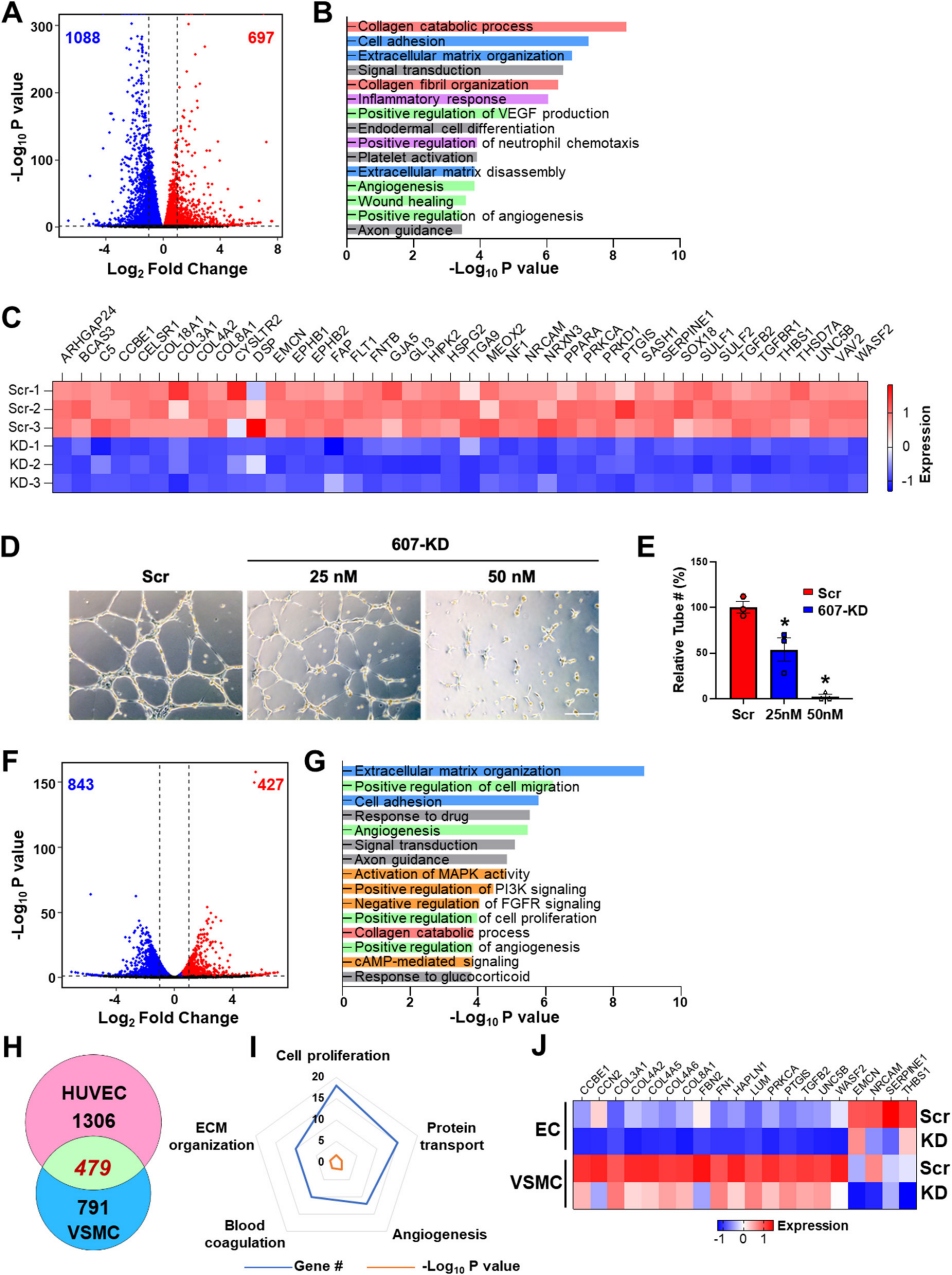
LINC00607 regulates basal EC and VSMC function. **(A)** Volcano plot indicating the DEGs upon 607-KD in HUVECs. Red and blue dots represent significantly up-regulated and down-regulated genes (with *P*-value cut-off of 0.01). Vertical dotted lines correspond to 2-fold differences. **(B)** Pathway enrichment analyses of DEGs upon 607-KD in HUVECs. Top 15 GO terms ranked by fold enrichment score for both the down- and up-regulated DEGs. **(C)** Expression of select down-regulated DEGs involved in angiogenesis due to LINC00607 knockdown (KD), as compared to scramble LNA (Scr). Heatmap is plotted based on z-scaled gene expression levels. **(D)** Representative images of tube formation of HUVEC transfected with either scramble or 607-KD at the indicated final concentration. The images were taken after incubating in Matrigel for 4 h. Scale bar = 100 μm. **(E)** Quantitative data of tube formation represented by numbers of tubes in randomly selected views. **(F)** DEGs upon 607-KD in VSMCs were plotted as in **(A)**. **(G)** Pathway enrichment analyses of DEGs upon 607-KD in VSMCs. Top 15 GO terms are plotted as in **(B)**. **(H)** Venn diagram showing the number of common DEGs upon 607-KD in HUVECs and VSMCs (in green). Numbers of unique DEGs in each cell type (pink for HUVECs and blue for VSMCs) are indicated. **(I)** Polygon radar chart showing the top 5 enriched pathways of the common DEGs in HUVECs and VSMCs. Data values for each vertex represent the number of DEGs classified in the indicated gene pathway in blue and the *P*-value (in -log_10_) in orange. **(J)** Expression heatmap of representative DEGs commonly affected by 607-KD in ECs and VSMCs.

Given the active transcription and SE state of *LINC00607* in both ECs and VSMC, and that SEs are important to maintain cell lineage and identity, we queried the role of LINC00607 in the regulation of basal function of ECs and VSMCs. To this end, we used a locked nucleic acid (LNA)-GapmeR to knockdown LINC00607 (607-KD) in HUVECs and HVSMC and performed RNA-seq simultaneously in the two cell types. Compared to the scramble control, LINC00607 inhibition led to 1,785 differentially expressed genes (DEGs) with 697 up- and 1,088 down-regulated genes in ECs (Figure 2A). Pathway enrichment analysis of these DEGs revealed that collagen catabolic process, extracellular matrix (ECM) organization, cell adhesion, inflammatory response, and angiogenesis are regulated by LINC00607 in ECs (Figure 2B). Specifically, DEGs involved in ECM organization, cell adhesion, and angiogenesis such as *COL4A1, CTGF, FN1, SERPINE1*, and *TGFB2* were significantly downregulated upon 607-KD in ECs (Figure 2C; Supplementary Figures 3B,E). Consistently, 607-KD decreased the tube forming activity of ECs in a dose-dependent manner (Figures 2D,E), which was particularly significant in the early time points (1–4 h) (Supplementary Figure 4).

With the same cutoff, RNA-seq revealed a total of 1,270 DEGs in VSMCs due to 607-KD, with 427 up- and 843 down-regulated (Figure 2F). Interestingly, while 607-KD affected similar pathways in VSMCs as in ECs, such as ECM organization, cell adhesion, and collagen catabolic process, it also affected different pathways in VSMCs, e.g., cell proliferation and c-AMP signaling (Figure 2G). ECs and VSMCs shared 479 common DEGs, comprising 26.8% in ECs and 37.7% of DEGs in VSMCs (Figure 2H). The top enriched GO terms of these DEGs included cell proliferation, protein transport, angiogenesis, ECM organization, and blood coagulation (Figures 2I,J). Of note, FN1, the key ECM component is downregulated in both ECs and VSMCs (Figure 2J), suggesting LINC00607 RNA participates in the *cis* regulation of *LINC00607* SE. On the other hand, the broad effect in other genes due to 607-KD suggests that LINC00607 RNA may regulate other genes through *trans* regulation, which may be independent from the SE function. Together, these data support an essential role for LINC00607 in the basal functions of these vascular cells, including angiogenesis and ECM organization.

### Stimulus-Dependent Regulatory Function of LINC00607 in ECs

**Figure 3:**
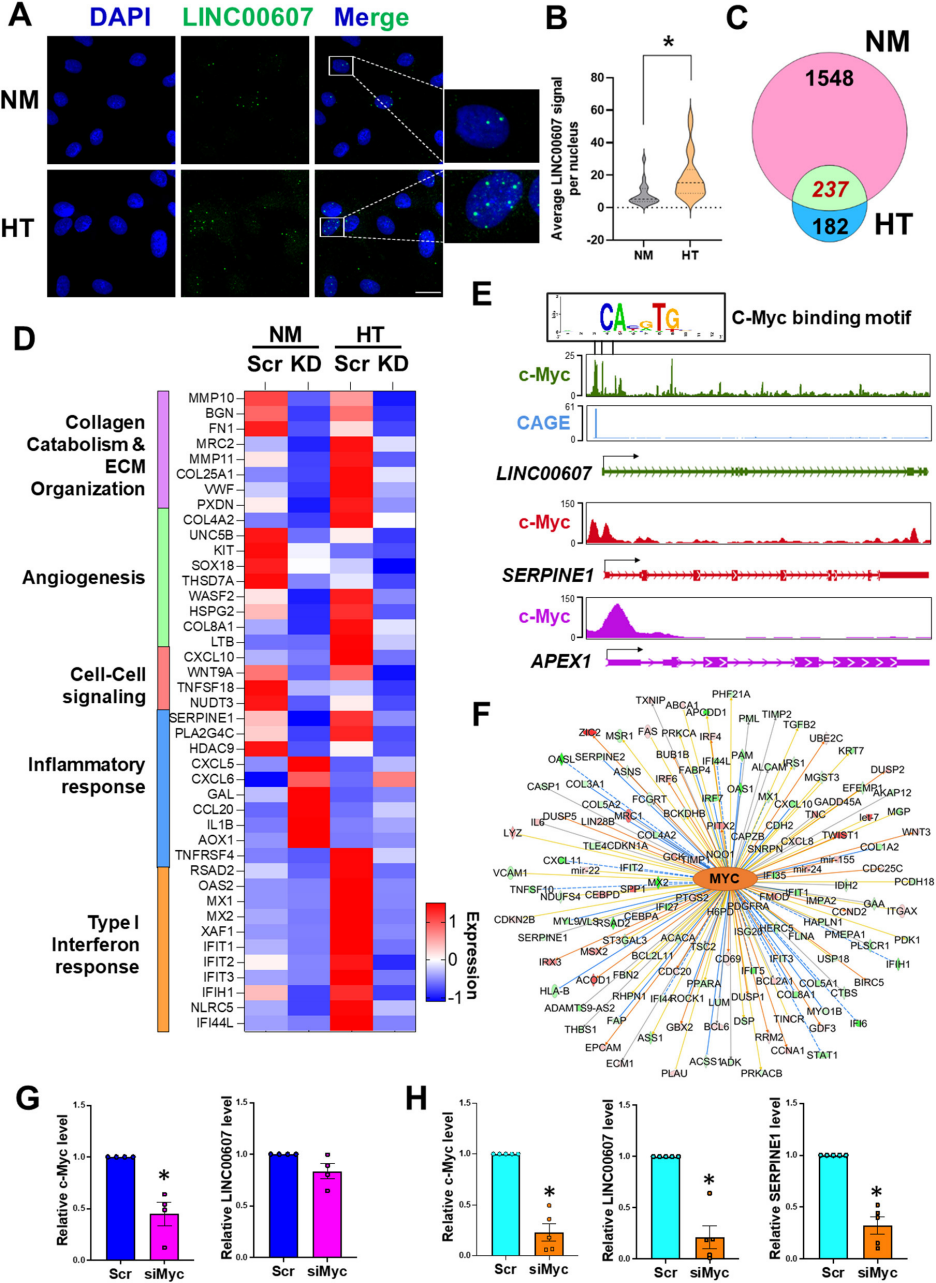
Stimulus-dependent regulatory function of LINC00607 in ECs. **(A)** Representative images of smFISH detecting LINC00607 (green) in HUVEC treated with normal glucose (5.5 mM D-glucose) and osmolarity control (NM) or 25 mM D-glucose and 5 ng/ml TNFα (HT) for 3 days. DAPI staining indicates the nuclei. Scale bar = 20 μm. **(B)** Quantitative data of LINC00607 signal per nucleus. LINC00607 signal from 18 nuclei for NM and 24 nuclei for HT was quantified using ImageJ. **(C)** Venn diagram showing the number of common DEGs in HUVECs upon 607-KD under basal and HT conditions (green). Numbers of unique DEGs in each condition (pink for NM and blue for HT) are indicated. **(D)** Expression pattern of select DEGs commonly regulated by 607-KD in HUVECs under both baseline and HT. Heatmap is plotted based on z-scaled average gene expression levels. **(E)** Putative sequence of c-Myc binding site at the LINC00607 promoters (Chr2: 215826423-215837423, Chr2: 215830609-215841609, and Chr2: 215842628-215853628) predicted by TRANSFAC. The HUVEC c-Myc ChIP-seq tracks (ENCODE data ENCFF000RUU) at LINC00607 (green), SERPINE1 (red), and APEX1 (purple) genomic loci were plotted using Epigenome Browser. CAGE tracks indicating the putative alternative TSS for LINC00607 is shown in blue **(F)** Network map showing MYC as a top candidate TF for LINC00607-regulated DEGs. The relationship between MYC and downstream targets are displayed as edges between nodes. The color intensity of each node represents fold change expression, red (upregulated), and green (downregulated). The edges denote predicted relationships with orange indicating activation, blue indicating inhibition and gray representing an unpredicted effect. **(G,H)** qPCR analysis of indicated transcripts in HUVECs transfected with respective siRNAs under basal condition **(G)** or HT **(H)**. The respective scramble control was set as 1. Data represents mean ± SEM from 3 independent experiments. *P < 0.05 based on *t*-tests between scramble and siMyc groups.

We previously demonstrated a pro-inflammatory and pro-fibrotic role of LINC00607 in ECs under HT (28). Given the profound regulation of basal EC function by LINC00607 shown in the present study (Figure 2), we hypothesized that LINC00607 is required for EC homeostasis, but that its induction by pathophysiological conditions, through aberrant regulation of the downstream genes, promotes EC dysfunction. Consistent with our previous data from RNA-seq and qPCR (28), we observed induction of LINC00607 by HT in ECs using smRNA FISH, which showed the primary localization of LINC00607 in the nucleus (Figures 3A,B). Moreover, there was a higher enrichment of H3K27ac signal on *LINC00607* locus in HT-treated ECs compared to NM control (Supplementary Figure 5), suggesting a more active SE state under HT. Comparison of DEGs identified from basal condition vs. HT-treated ECs revealed a total of 237 common DEGs (Figure 3C), which are involved in a series of intrinsic EC functions, including collagen catabolism and ECM organization, angiogenesis, cell-cell signaling, inflammatory response and type I interferon response (Figure 3D).

TFs play a central role in regulating the transcriptome by binding to promoters and enhancers, with the latter coordinating with the TFs to regulate its transcriptional targets (16, 46, 47). To gain insights into the upstream regulator of *LINC00607* and the downstream transcriptional network regulated by LINC00607 RNA, we performed two analyses: (1) TRANSFAC analysis to predict potential TFs that can regulate LINC00607 transcription through binding its promoter; and (2) Ingenuity Pathway Analysis (IPA) to discover TFs that may coordinate with LINC00607 to regulate the DEGs affected by 607-KD (i.e., putative LINC00607 targets) in ECs. Integrating the two analyses revealed c-Myc to be a top candidate TF. Examining the c-Myc ChIP-seq data from HUVEC available in ENCODE and Epigenome Browser, we found specific Myc-binding signals at the promoter regions of *LINC00607*, which partially match the predicted c-Myc TF binding sites (Figure 3E). In the same dataset, c-Myc also shows clear binding to *SERPINE1* and *APEX1*, two known targets of c-Myc (48, 49) (Figure 3E). IPA analysis independently revealed c-Myc as a top candidate upstream TF of LINC00607-regulated DEGs ranked by the number of target genes and activation scores (Figure 3F; Supplementary Table 3).

To further test whether c-Myc regulates LINC00607, we knocked down c-Myc in HUVECs by small interference RNAs (siRNAs) as confirmed by immunoblotting (Supplementary Figure 6). While c-Myc inhibition did not significantly affect the expression of LINC00607 under basal conditions (Figure 3G), there was a strong reduction in LINC00607 expression upon c-Myc inhibition under HT (Figure 3H). Consistently, the expression of SERPINE1, a proven regulatory target of LINC00607 and c-Myc, was also downregulated upon c-Myc inhibition (Figure 3H). Taken together, c-Myc appears to be a TF controlling the expression of LINC00607, which may act as an integral regulator in the c-Myc transcriptional network especially in the dysfunctional ECs.

### LINC00607 Inhibition Partially Reverses HT-Induced Endothelial Transcriptomic Profile

**Figure 4:**
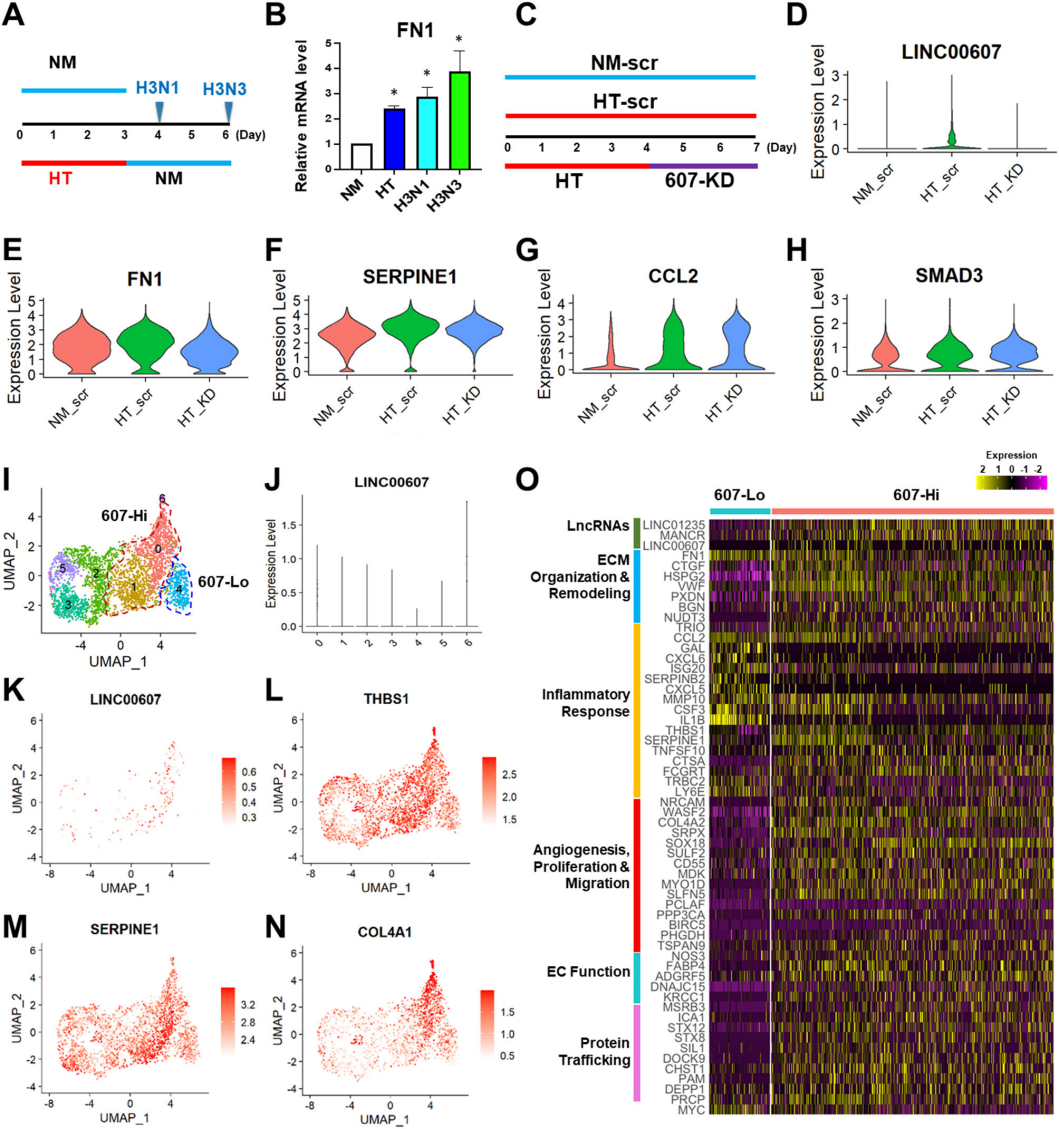
LINC00607 inhibition reverses the effect of HT in ECs. **(A)** Experimental design of HT to NM switch experiment. HUVECs were treated with NM or HT for 3 days, and then switched to NM medium for another 1 (H3N1) or 3 days (H3N3) before cell harvest. **(B)** qPCR detection of FN1 mRNA levels from experiment shown as in **(A)**. **(C)** Experimental design of the “reverse” experiment. HUVECs in biological replicates were treated with NM or HT for 7 days, or HT for 4 days before LINC00607 knockdown using LNA GapmeR (607-KD). **(D)** LINC00607 expression quantified by scRNA-seq of three groups of ECs. **(E–H)** Representative reversible (*FN1* and *SERPINE1*) and irreversible (*CCL2* and *SMAD3*) DEGs. **(I)** UMAP of HT-KD scRNA-seq data. **(J)** LINC00607 expression in HT-KD samples shown on UMAP separated by 7 clusters. Note that Cluster 0, 1, and 6 show high LINC00607 levels (expression level >1, i.e., 607-Hi cells) and Cluster 4 shows the lowest LINC00607 level (i.e., 607-Lo cells). **(K)** LINC00607 expression in 7 clusters of cells in HT-KD samples plotted by UMAP. **(L–N)** Representative DEGs in HT-KD samples plotted on UMAP. **(O)** Heatmap showing the expression of indicated DEGs in LINC00607-Lo vs. -Hi ECs.

It has been well-recognized that metabolic dysfunction associated with diabetes can provoke sustained alterations that contribute to persistent organ damage. In ECs, DNA methylation and histone modifications have been implicated in the sustained effect of hyperglycemia (5, 50, 51). We previously observed that whereas the HT-induced changes of some gene expression, such as eNOS and ICAM1 can be reversed by switching to normoglycemia, LINC00607 and its regulated SERPINE1 cannot be reversed (28). In line with this finding, HT-induced *FN1* expression also continued to increase even after the replacement of high glucose with normal glucose (Figures 4A,B). Thus, we asked whether LINC00607 mediates the prolonged effect of high glucose and if so, whether its inhibition can reverse the effect of HT in ECs. To this end, we altered our approach of 607-KD prior to HT treatment (as we did for bulk RNA-seq shown in Figure 3), to instead exposing HUVECs to HT treatment for 4 days and then inhibiting LINC00607 for the next 3 days (Figure 4C). Because the transfection efficiency is unlikely to be 100%, we performed scRNA-seq analysis to resolve the cell heterogeneity of responses. We sequenced 2,806, 1,846 and 3,407 cells in three treatment groups, i.e., ECs kept under NM and transfected with scramble LNA (NM-scr), EC kept under HT and transfected with scramble LNA (HT-scr), and ECs kept under HT first and then transfected with LINC00607 LNA (HT-KD), respectively. The HT induction of LINC00607 and inhibition of LINC00607 was evident by scRNA-seq (Figure 4D). However, UMAP did not reveal the 3 groups of ECs as separated clusters (Supplementary Figure 7A), suggesting that ECs remain largely same population despite the treatments. To identify the specific genes whose expression is altered by HT and attenuated/reversed by 607-KD, we identified DEGs between NM-scr vs. HT-scr and those between HT-scr and HT-KD. Such analysis revealed 60 DEGs (Supplementary Figures 7B,C). This list of reversible genes includes *FN1, SERPINE1*, and *THBS1*, all of which were also downregulated by 607-KD in the bulk RNA-seq (Figures 4E,F; Supplementary Figures 7C,E). On the other hand, such analysis also identified 67 genes that were irreversible upon 607-KD such as *CCL2, SMAD3*, and *ICAM1* (Figures 4G,H; Supplementary Figures 7D,F).

Next, we focused on the HT-KD samples to dissect the effect of LINC00607 inhibition in single ECs. Based on LINC00607 levels, the ~3,000 ECs were separated into 7 clusters (Figure 4I), indicating the expected heterogeneity in the transfected ECs. Cluster 6 exhibited the highest abundance of LINC00607, followed by Clusters 0 and 1. In contrast, Cluster 4 exhibited the lowest LINC00607 level (Figures 4I–K). Differential expression analysis of the 3 clusters with highest LINC00607 levels (hereafter called 607-Hi cells) relative to Cluster 4 (called 607-Lo cells) identified 609 DEGs including *SERPINE1, THBS1*, and *COL4A1*, which were also 607-KD-reversible genes (Figures 4L–N). In line with the bulk RNA-seq data in which 607-KD was performed prior to HT treatment, 607-Lo cells also expressed lower levels of various pro-inflammatory, pro-ECM remodeling, and pro-fibrotic genes (Figure 4O). These data support that ECs with low LINC00607 levels, most likely due to LINC00607 inhibition, are also ECs with LINC00607-downstream genes suppressed, corroborating a positive role of LINC00607 in the sustained effect of HT.

## Discussion

SEs play a major role in controlling cell identity and homeostasis (20, 21). It is plausible that SE-derived lncRNAs participate in the SE-regulated cell functions. Given the active SE and transcriptional state of *LINC00607* in ECs and VSMCs, we postulated that this same lncRNA may regulate shared and differential functional aspects in these two distinct but closely related cell types in the vasculature. In support of this hypothesis, we observed that inhibition of LINC00607 affects ~30% DEGs common to ECs and VSMCs, which are involved in ECM organization, collagen catabolism, and angiogenesis, suggesting an overall fundamental role for LINC00607 in vascular physiology. In both cell types, 607-KD decreased the nearby FN1 expression, suggesting a shared *cis* regulatory role for *LINC00607*, which involved the SE-derived lncRNA. Moreover, LINC00607 inhibition also leads to profound changes of many genes encoded far away from *LINC00607*, supporting the *trans* regulation by LINC00607 RNA, consistent with our previous observation (28). Specific to each cell type, LINC00607 regulates primary EC functions including angiogenesis, VEGF production, and wound healing and major VSMC functions such as cell proliferation and migration.

Aside from the various pathways essential for normal EC and VSMC functions, genes involved in pathological pathways and vascular dysfunction such as inflammatory response, fibrosis, and ECM remodeling were also differentially regulated upon 607-KD in ECs and VSMCs, pointing toward a potential role for this lncRNA in disease states. Along these lines, we previously found that inhibition of LINC00607 in diabetes-mimicking HT condition significantly suppressed endothelial inflammatory response as evidenced by suppression of monocyte adhesion to ECs (28). In VSMCs, we also observed an induction of LINC00607 by Angiotensin II (AngII) treatment (Supplementary Figure 8) and our unpublished observation shows that 607-KD reduces AngII-induced *FN1* expression. Thus, LINC00607 represents another AngII-induced lncRNA in VSMC along with those previously reported by us (31, 52). Considering the earlier study demonstrating the activation of SEs by TNFα to promote inflammation and atherogenesis (23), our previous work showing the induction of SE hubs for RNA-chromatin interactions by HT to contribute to endotheliopathy (28), and that AngII-induced signals integrate enhancers/SEs and lncRNAs to promote VSMC dysfunction (53), our current findings argue that whereas SEs are key to cell lineage and identity, the overactivation of SEs, in part through SE-derived lncRNAs may drive cell fate toward disease state.

TFs are quintessential in the regulation of lncRNAs and their target genes. While the TF c-Myc has been reported to regulate the expression of several lncRNAs in cancers, none have been reported in ECs (54–56). Based on *LINC00607* genomic sequence as well as LINC00607 regulatory gene network, we identified c-Myc to be a common upstream transcriptional regulator of *LINC00607*, which may be an integral element coordinating with c-Myc for transcriptional control of ECs. Interestingly, inhibition of c-Myc suppressed the levels of LINC00607 under HT but not under baseline conditions. This suggests that c-Myc may act as a stimulus-dependent TF to activate LINC00607 transcription under a pathophysiological state such as diabetes. However, under basal condition, other TFs including lineage-dependent TFs may play a major role in controlling the SE transcriptional state (21). Indeed, one key EC-specific LDTF, i.e., KLF2/4 was also predicted as an upstream TF of LINC00607 gene network (data not shown). On the other hand, alternative or additional TFs may coordinate with c-Myc to regulate LINC00607 in other cell contexts. For example, from our TRANSFAC analysis, we found MYC heterodimer with the MYC-associated factor X (MAX) as a putative upstream TF identified to bind LINC00607 promoter regions. Moreover, AP-1/JUN, a major AngII-responsive TF that regulates FN1 (53), was also among the highly ranked TFs upstream of LINC00607 target network (Supplementary Table 3). The molecular mechanisms of *LINC00607* regulation in different cell types and under different cell states, and the mechanism by which LINC00607 further regulates its downstream targets in these different contexts are of interest for future studies.

To probe the question whether SE-lncRNAs are involved in the prolonged effect of high glucose, we performed the “reversal experiment,” i.e., treating ECs with HT and then inhibiting LINC00607 when EC dysfunction was in-place. Although this is a purely *in vitro* system, it imitates to a degree the conditions found in diabetic patients, who may already be hyperglycemic and undergoing diabetes-induced endotheliopathy before treatment. It is intriguing that 607-KD could reverse or attenuate HT induction of some genes, e.g., *FN1* and *SERPINE1*, whose induction by HT could not be reversed by switch to normal glucose. In contrast, 607-KD failed to do so for other genes, such as ICAM1, whose induction by HT could be reversed by switching to normal glucose (28). These findings suggest that chromatin-associated lncRNAs exemplified by LINC00607, perhaps by impacting the nuclear organization and function, contribute, at least in part, to the sustained transcriptional and phenotypic changes underlying the persistent organ damage despite glucose normalization. This may provide an additional layer of epigenetic mechanism, other than DNA methylation and histone modification, to explain the metabolic memory and legacy effect observed in diabetic patients who continue to develop complications even after glucose normalization (5). From a therapeutic point of view, interventions that target both hyperglycemia and SE-derived lncRNAs, e.g., LINC00607, may be more effective than glucose control alone. Future studies are warranted to identify other lncRNAs that contribute to the epigenetic memory and develop therapeutic strategies to ameliorate the diabetes-induced cellular changes through targeting SE-lncRNAs.

## Data Availability Statement

The datasets presented in this study can be found in online repositories. The names of the repository/repositories and accession number(s) can be found below: GEO, GSE197956.

## Ethics Statement

The studies involving human participants were reviewed and approved by Institutional Review Board of City of Hope (IRB #01046). The research consents for the use of human donor tissues were obtained from the donors' next of kin.

## Author Contributions

YL, RN, and ZBC: conceived the research. YL, VAS, RN, and ZBC: designed the experiments. YL, KS, AT, and VAS: performed the experiments. YL, KS, DY, NM, and AT: analyzed and plotted data. ZBC and RN: supervised experiments and interpreted results. YL, KS, NM, RN, and ZBC: wrote the manuscript. RN and ZBC: obtained funding for this study. All authors contributed to the article and approved the submitted version.

## Funding

This study was in part funded by grants from the National Institutes of Health (NIH) (R01 HL145170 to ZBC, and R01HL106089 to RN and ZBC, R01 DK065073 and R01 DK081705 to RN), a Human Cell Atlas seed network grant from Chan Zuckerberg Foundation (to ZBC) and Ella Fitzgerald Foundation (to ZBC), and an American Heart Association Pre-doctoral fellowship (to VAS). Research reported in this publication included work performed in the Integrative Genomics Core at City of Hope supported by the National Cancer Institute of the National Institutes of Health under award number P30CA033572.

## Conflict of Interest

The authors declare that the research was conducted in the absence of any commercial or financial relationships that could be construed as a potential conflict of interest.

## Publisher's Note

All claims expressed in this article are solely those of the authors and do not necessarily represent those of their affiliated organizations, or those of the publisher, the editors and the reviewers. Any product that may be evaluated in this article, or claim that may be made by its manufacturer, is not guaranteed or endorsed by the publisher.
