# Supplementary material for: Vascular Regulation by Super Enhancer-Derived LINC00607
Source: Front Cardiovasc Med. 2022 Jun 28;9:881916. doi: 10.3389/fcvm.2022.881916 (PMC9274098; doi:10.3389/fcvm.2022.881916)
Supplement: Supplementary file 1 [file Data_Sheet_1.pdf]

Supplementary Figures and Tables

Supplementary Figure 1. smFISH of mesenteric artery from healthy and diabetic human donors with a negative control probe. DAPI staining indicative of nuclei. Scale bar = 100µm.

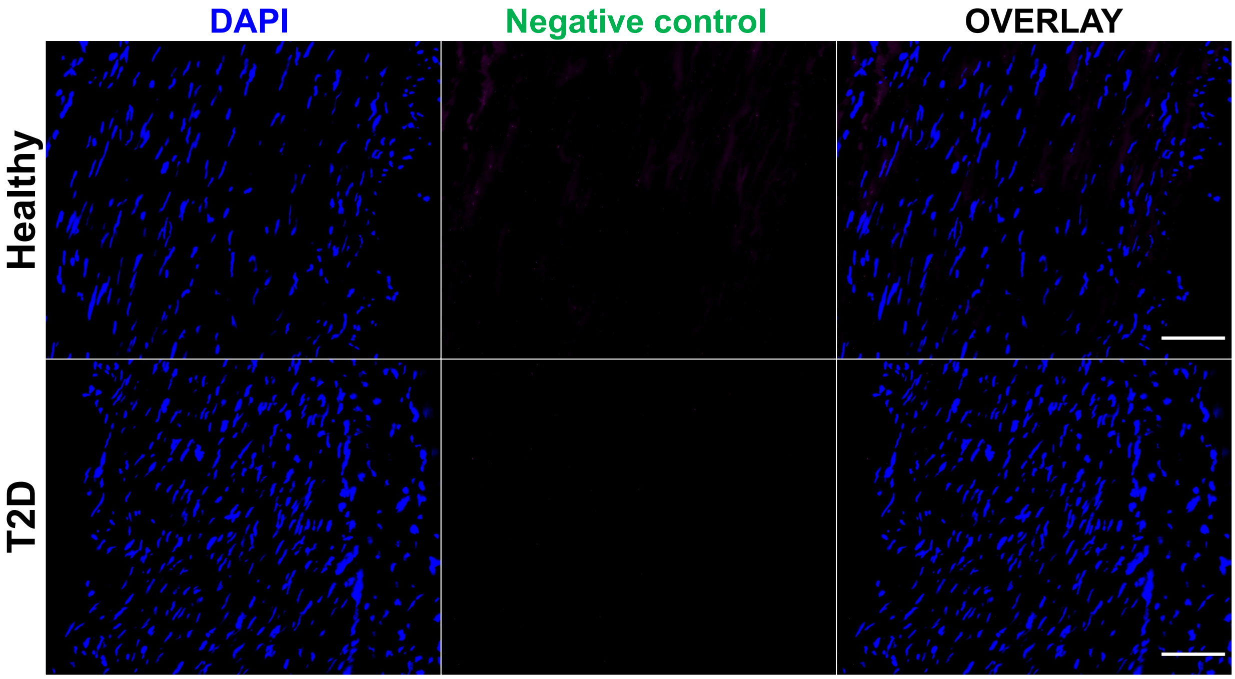

Supplementary Figure 2. Expression of LINC00607 and other lncRNAs shown to be important in EC or VSMC. Heatmap plotted from 43 samples of 22 primary human cell types retrieved from ENCODE project.

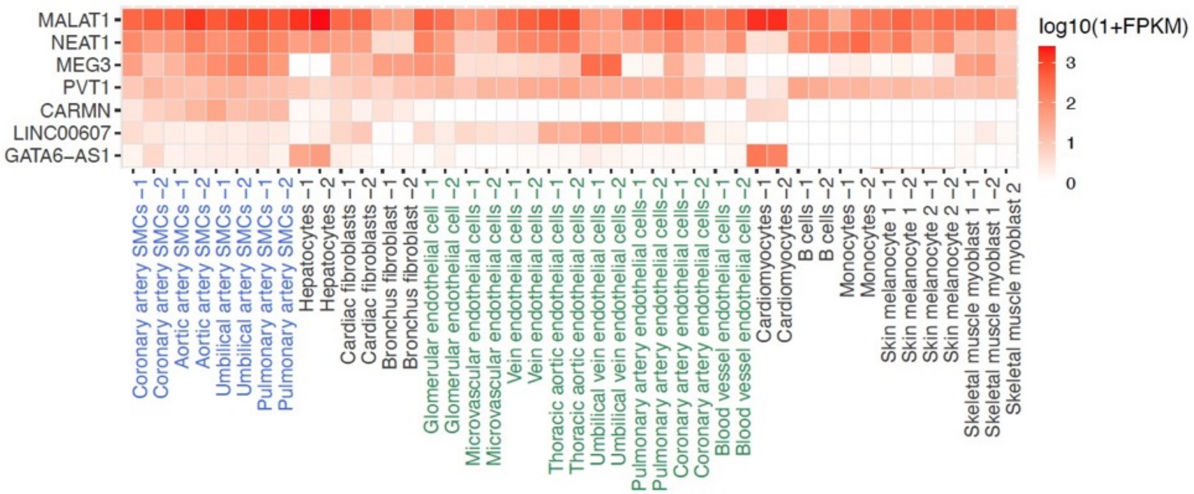

Supplementary Figure 3. Pathway enrichment analyses of up- or down-regulated genes by 607-KD in HUVECs **(A and B)** or VSMCs **(C and D)**. Top 10 Gene Ontology (GO) terms ranked by fold enrichment score are plotted. **(E)** mRNA levels of select EC dysfunction markers were quantified by RT-qPCR in HUVECs transfected with scramble or LINC00607 LNA. Data presented as mean ± SEM from 3 independent experiments. * denotes P value < 0.05.

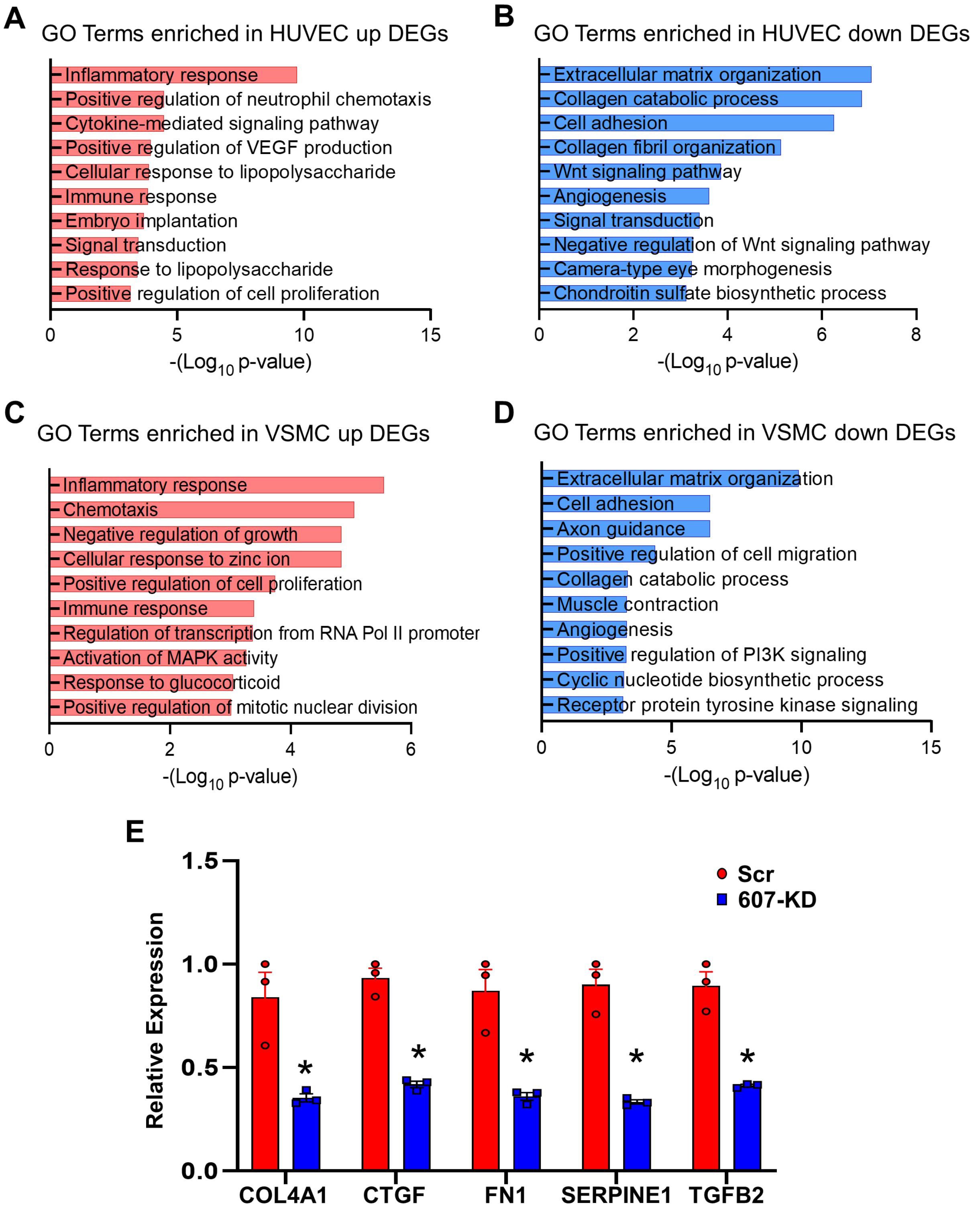

Supplementary Figure 4. (A) Representative images of tube formation of HUVEC transfected with either scramble LNA (Scr) or LNA targeting LINC00607 (607-KD). The images were taken after plating the cells on Matrigel for 2, 4, 8 and 14 hours as indicated. Scale bar = 400 µm. **(B)** Quantification of number of tubes formed in indicated conditions from randomly selected views. Data presented as mean ± SEM from 3 independent experiments.

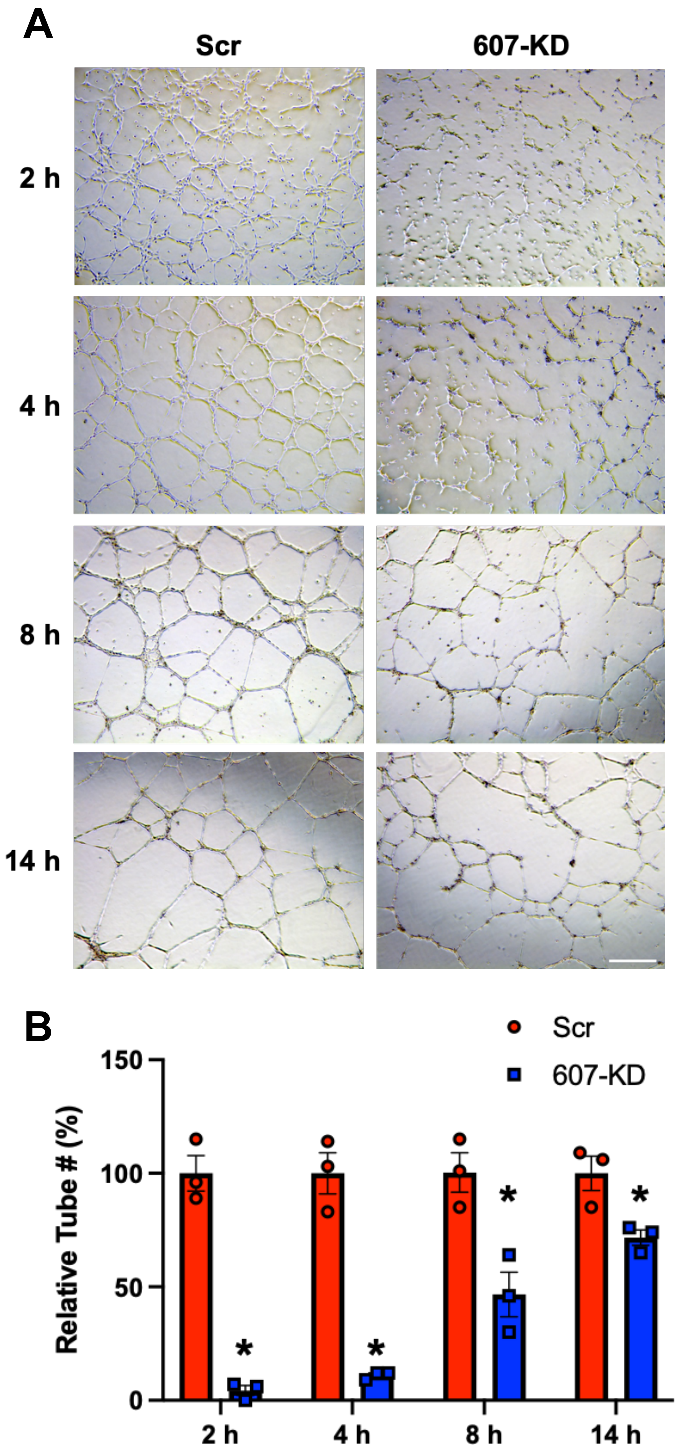

Supplementary Figure 5. Enrichment of H3K27ac on LINC00607 genomic locus in HUVECs treated with mannitol (NM) or 25 mM D-glucose and 5 ng/ml TNFα for 3 days (HT) determined by ChIP-qPCR. The enrichment level under NM was set as 1. Data are presented as mean ± SEM. * denotes $P < 0.05$ based on Mann-Whitney test between the two conditions.

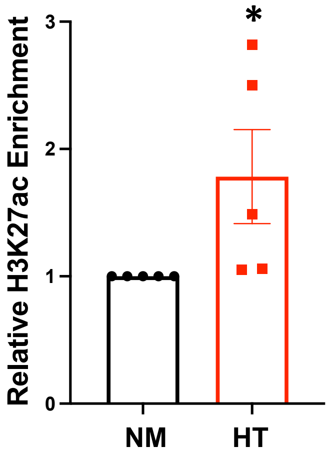

Supplemental Figure 6. (A-C) HUVECs were transfected with pool of siRNAs targeting c-Myc at 20 nM (siMyc) or control siRNA (siCtrl) in biological replicates. Immunoblotting was performed with indicated antibodies to detect c-Myc protein or β-actin.

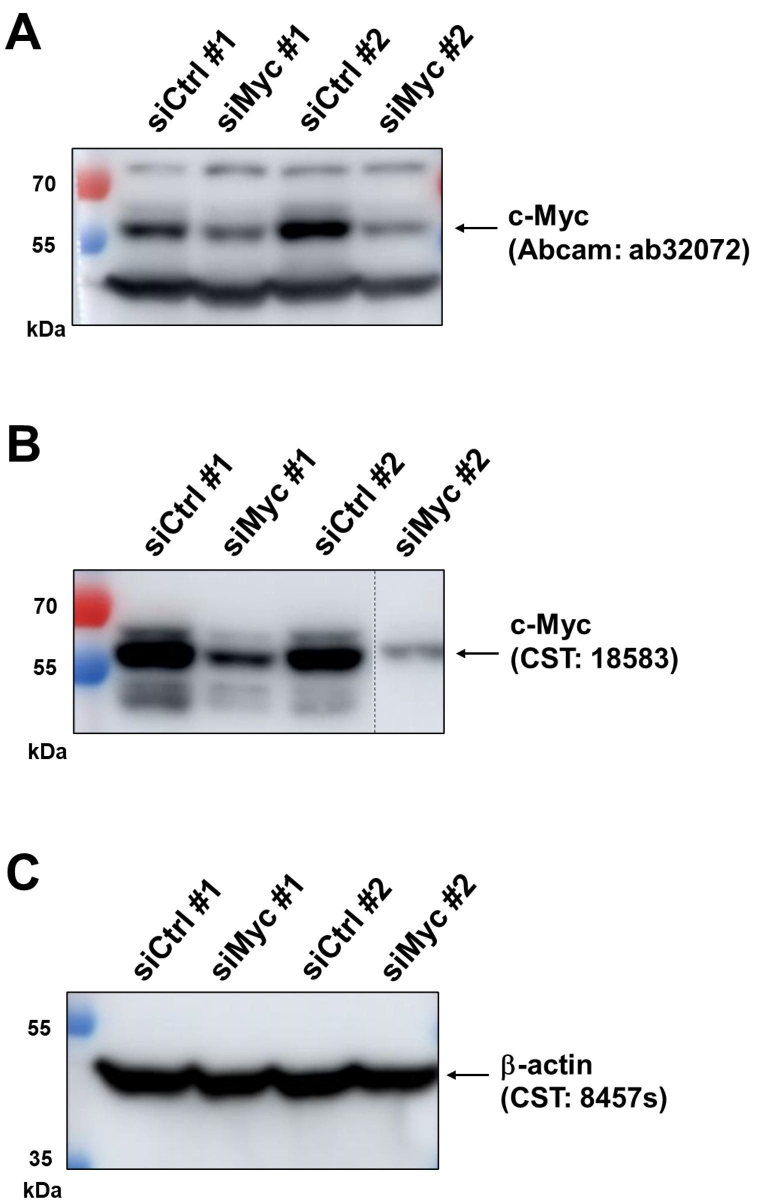

Supplementary Figure 7. (A) UMAP of scRNA-seq samples showing the ECs largely remain the same population after the indicated treatments. **(B)** Venn-diagram showing genes reversible by 607-KD i.e., genes that are induced by HT and suppressed upon 607-KD (41 genes), and genes that are suppressed by HT and induced upon 607-KD (19 genes). **(C)** Heatmap of genes that are altered by HT and reversed upon 607-KD (reversible genes). **(D)** Heatmap of genes that are altered by HT but are not affected upon 607-KD (irreversible genes). **(E, F)** Expression pattern of FABP4 (a reversible gene) and ICAM1 (an irreversible gene) in the different treatment groups.

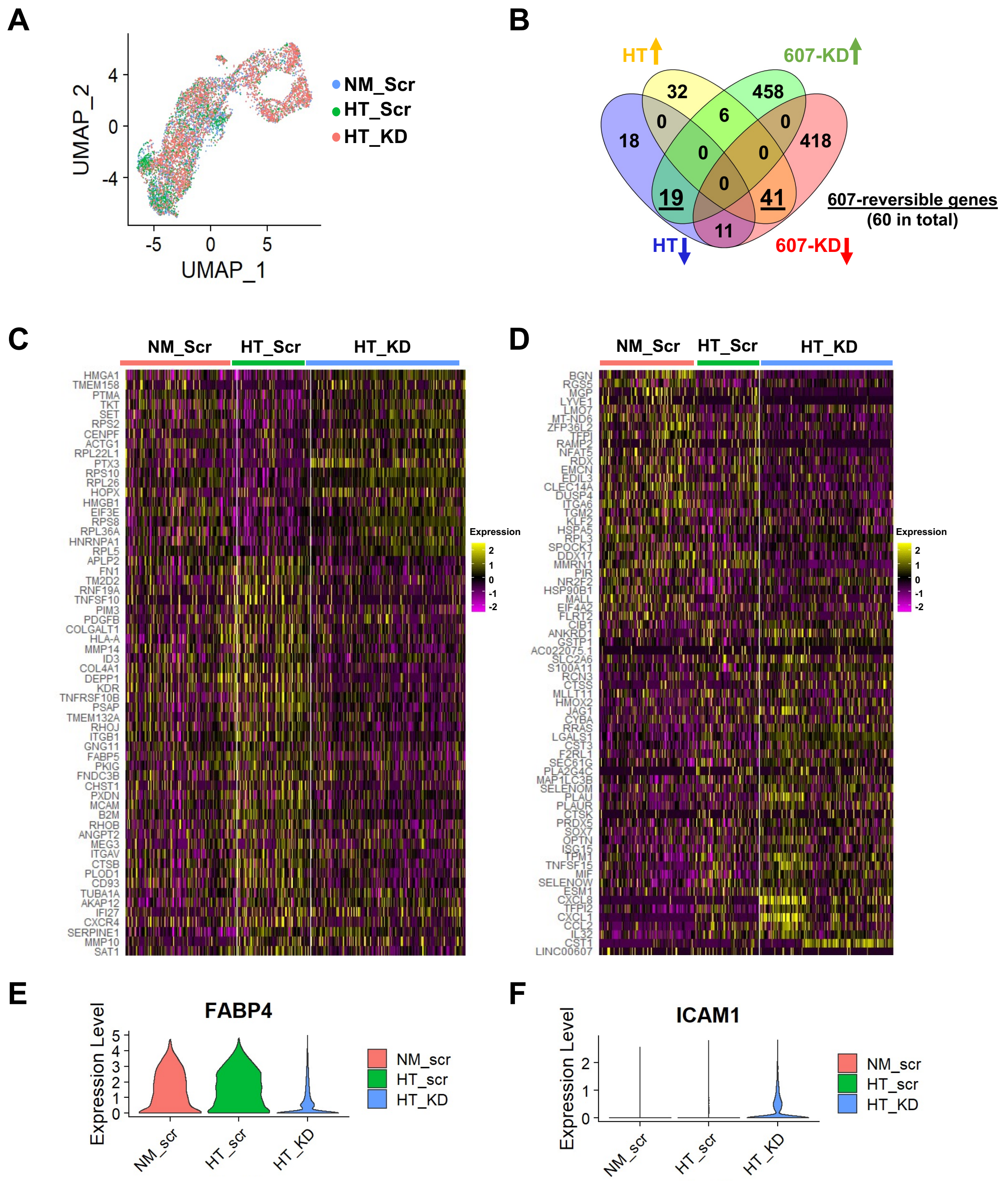

Supplementary Figure 8. RNA levels of LINC00607 **(A)** and FN1 **(B)** in VSMC treated with control (Ctrl) or Angiotensin II (100 nM) for 12 hours or 24 hours as determined by qPCR. Data presented as mean ± SEM. * denotes $P < 0.05$ based on unpaired t test relative to control.

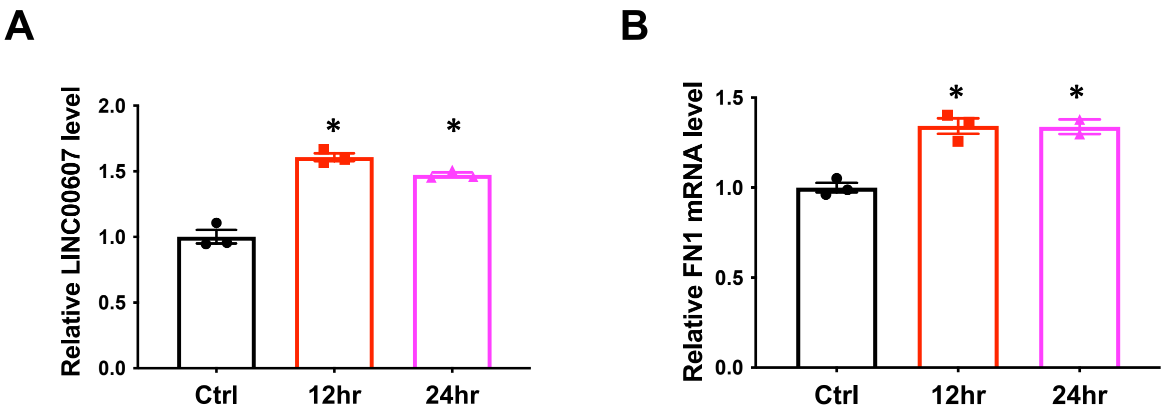

Supplementary Table 1. Donor information

Variable	HC		T2D	
Sex	Male	Female	Male	Female
Number	6	0	6	2
*Age, y	41.8±2.6		44.8±3.7	
*BMI, kg/m^2	25.6±1.7		31.3±2.2	
*HbA1c, %	5.2±0.1		7.6±0.3	

Values represent Mean ± SEM. *denotes values that are of significant statistical difference between healthy and T2D donors.

Supplemental Table 2 Primers, LNA, and siRNA sequences

Primers	Forward	Reverse
**LINC00607 RNA**	ACCGGGCGTTGAGAATACAA	ACACTTGGCGAAACTTCCCT
**c-Myc mRNA**	GCTGCTTAGACGCTGGATTT	CTCCTCCTCGTCGCAGTAGA
**ACTB mRNA**	CATGTACGTTGCTATCCAGGC	CTCCTTAATGTCACGCACGAT
**SERPINE1 mRNA**	AGTGGACTTTTCAGAGGTGGA	GCCGTTGAAGTAGAGGGCATT
***LINC00607* ChIP**	ATGTTTGCCTTGGAAGGTTG	GCCCTCTCACCCTAAAAACC

LNA GapmeRs	Sequence	Position (NR_037195.1)
**LINC00607**	ATAGGTCACGCATTCT	210-225
**Scramble**	AACACGTCTATACGC	

MYC siRNA ID	Order No.	Sequence
**s9129**	4427038	ATCAAAAACATCATCATCCAG
**s9130**	4427039	CTGGTCCTCAAGAGGTGCCAC
**s9131**	4427040	ACGGAGCTTTTTGCCCTGCG

Supplementary Table 3. Top 20 TFs of LINC00607 regulatory network predicted by IPA

Upstream Regulator	Predicted Activation State	Activation z-score	P value of Overlap	# Target Molecules
TP53		-0.591	5.99E-15	226
HNF4A		-1.932	0.256	157
MYC	Activated	2.058	9.99E-09	144
STAT3	Activated	4.097	1.07E-29	136
CTNNB1		0.073	2.67E-09	121
STAT1		-1.642	3.73E-36	111
SMARCA4		0.094	1.29E-17	106
SP1		-0.464	1.92E-15	95
JUN	Activated	2.405	2.13E-17	91
FOS		1.516	3.9E-11	85
STAT6	Activated	3.414	2.49E-15	82
CREB1	Activated	3.396	1.3E-09	80

GLI1		1.586	2.45E-09	75
SIRT1		1.966	1.5E-12	75
NKX2-3	Activated	2.97	5.01E-32	74
RELA		1.915	4.93E-11	74
CEBPB	Activated	3.051	7.89E-06	72
HTT		-0.134	0.00389	72
CEBPA		1.812	5E-11	72
SOX2		1.161	4.53E-09	71

Supplementary Table 4. Summary of sequencing data

Accession ID	Sample Description
GSE197956	Vascular Regulation by Super Enhancer-Derived LINC00607
GSE197955	Vascular Regulation by Super Enhancer-Derived LINC00607 (scRNA-Seq)
GSM5934492	HUVEC_Scr_NM_1
GSM5934493	HUVEC_Scr_HT_1
GSM5934494	HUVEC_LNA_HT_1
GSM5934495	HUVEC_Scr_NM_2
GSM5934496	HUVEC_Scr_HT_2
GSM5934497	HUVEC_LNA_HT_2
GSE197954	Vascular Regulation by Super Enhancer-Derived LINC00607 (bulk RNA-Seq)
GSM5934482	HUVEC_Scr1
GSM5934483	HUVEC_Scr2
GSM5934484	HUVEC_Scr3
GSM5934485	HUVEC_607KD1
GSM5934486	HUVEC_607KD1
GSM5934487	HUVEC_607KD1
GSM5934488	HVSMC Scr1
GSM5934489	HVSMC 607KD1
GSM5934490	HVSMC Scr2
GSM5934491	HVSMC 607KD2
